# Epigenetics, heritability and longitudinal analysis

**DOI:** 10.1186/s12863-018-0648-1

**Published:** 2018-09-17

**Authors:** Haakon E. Nustad, Marcio Almeida, Angelo J. Canty, Marissa LeBlanc, Christian M. Page, Phillip E. Melton

**Affiliations:** 1 Department of Medical Genetics, Oslo University Hospital, Kirkeveien 166, 0450 Oslo, Norway; 2 Faculty of Medicine, University of Oslo, Klaus Torgårds vei 3, 0372 Oslo, Norway; 3 PharmaTox Strategic Research Initiative, University of Oslo, Sem Sælands vei 3, 0371 Oslo, Norway; 4 South Texas Diabetes and Obesity Institute, University of Texas Rio Grande Valley School of Medicine, One West University Blvd., STDOI Modular Building #100, Brownsville, TX 78520 USA; 5 Department of Mathematics and Statistics, McMaster University, 1280 Main St. W, Hamilton, ON L8S 4K1 Canada; 6 Oslo Centre for Biostatistics and Epidemiology, Oslo University Hospital, Klaus Torgårds vei 3, 0372 Oslo, Norway; 7 Department of Non-communicable disease, Norwegian Institute of Public Health, Marcus Thranes Gate 6, 0473 Oslo, Norway; 8 Curtin/UWA Centre for Genetic Origins of Health and Disease, School of Pharmacy and Biomedical Sciences, Curtin University and the University of Western Australia, 35 Stirling Hwy. (M409), Crawley, WA 6009 Australia

**Keywords:** Epigenetics, Heritability, DNA methylation, Repeated measurements, Linear mixed effect models, Bayesian, Variance components

## Abstract

**Background:**

Longitudinal data and repeated measurements in epigenome-wide association studies (EWAS) provide a rich resource for understanding epigenetics. We summarize 7 analytical approaches to the GAW20 data sets that addressed challenges and potential applications of phenotypic and epigenetic data. All contributions used the GAW20 real data set and employed either linear mixed effect (LME) models or marginal models through generalized estimating equations (GEE). These contributions were subdivided into 3 categories: (a) quality control (QC) methods for DNA methylation data; (b) heritability estimates pretreatment and posttreatment with fenofibrate; and (c) impact of drug response pretreatment and posttreatment with fenofibrate on DNA methylation and blood lipids.

**Results:**

Two contributions addressed QC and identified large statistical differences with pretreatment and posttreatment DNA methylation, possibly a result of batch effects. Two contributions compared epigenome-wide heritability estimates pretreatment and posttreatment, with one employing a Bayesian LME and the other using a variance-component LME. Density curves comparing these studies indicated these heritability estimates were similar. Another contribution used a variance-component LME to depict the proportion of heritability resulting from a genetic and shared environment. By including environmental exposures as random effects, the authors found heritability estimates became more stable but not significantly different. Two contributions investigated treatment response. One estimated drug-associated methylation effects on triglyceride levels as the response, and identified 11 significant cytosine-phosphate-guanine (CpG) sites with or without adjusting for high-density lipoprotein. The second contribution performed weighted gene coexpression network analysis and identified 6 significant modules of at least 30 CpG sites, including 3 modules with topological differences pretreatment and posttreatment.

**Conclusions:**

Four conclusions from this GAW20 working group are: (a) QC measures are an important consideration for EWAS studies that are investigating multiple time points or repeated measurements; (b) application of heritability estimates between time points for individual CpG sites is a useful QC measure for DNA methylation studies; (c) drug intervention demonstrated strong epigenome-wide DNA methylation patterns across the 2 time points; and (d) new statistical methods are required to account for the environmental contributions of DNA methylation across time. These contributions demonstrate numerous opportunities exist for the analysis of longitudinal data in future epigenetic studies.

## Background

Longitudinal studies and repeated measurements in epigenome-wide DNA methylation studies (EWAS) can potentially provide insight into time- or condition-varying effects. Analysis where each subject serves as their own control allows for the assessment of within-individual variations over time and identification of factors associated with these time-dependent changes. Although repeated measure studies may be expensive to initiate and difficult to maintain, cross-sectional studies cannot detect the dynamic nature of epigenetic mechanisms impacting complex disease, making it difficult to ascertain whether the underlying causal effect is environmental or genetic [1]. Additionally, repeated measurements typically increase statistical power compared to cross-sectional designs.

Epigenetics is the study of reversible, mitotically heritable changes that influence gene control but do not alter the underlying DNA sequence. The most studied epigenetic mark is DNA methylation, which is a chemical process where a methyl group is added to the cytosine base, at a cytosine-phosphate-guanine (CpG) site, to form 5-methylcytosine. DNA methylation has been extensively studied in relation to diseases [2], where the focus lies on the discovery of differentially methylated CpG sites or regions. DNA methylation is influenced by age [3] and gender [4], along with environmental factors such as diet [5] and smoking [6].

Longitudinal studies are clearly beneficial for understanding how the epigenome changes over time and its involvement in the progression of complex disease etiology or response to change in condition, such as treatment [1]. Longitudinal analysis is also important when assessing epigenetic changes in heritability over the life course [7, 8]. Change in heritability over time might be an important feature of biological processes or adaptations to changing environments, and such interrogations can help with identifying environmental versus genetic contributions. Comparison of heritability estimates over several time points also provides an indication of consistency, which either can improve the certainty of the estimates or help identify technical issues with the epigenetic study.

Narrow-sense heritability (h^2^) is the proportion of phenotypic variance due to additive genetic variance. Traditionally, h^2^ is estimated using twins or parent–offspring pairs/trios, but other approaches using linear mixed effect models have been developed to estimate h^2^ in pedigrees of any size [9, 10]. These methodologies are well established for phenotypes possessing moderate to high h^2^ [10, 11], but are less well established for traits having low h^2^, as is expected for some proportion of CpG sites [12, 13]. Modifications in the epigenome, including DNA methylation, can alter gene expression in a heritable manner without impacting the underlying gene sequence. This model of epigenetic inheritance can be explained through (a) mitotic inheritance of phenotypes across cell generations, (b) inheritance across successive meiotic divisions, and (c) transgenerational inheritance, which requires proof of h^2^ across multiple generations [14].

The GAW20 provided data from individuals in up to 3-generation pedigrees. This provided the opportunity to investigate h^2^ of different traits, including metabolic syndrome, triglyceride (TG) levels and DNA methylation. In this paper, we summarize 7 GAW20 contributions (Table 1) focused on the development and application of statistical methodologies for the analysis of h^2^ and longitudinal DNA methylation data. Similar to previous GAW workshops, these contributions included a variety of statistical methods and strategies that dealt with the advantages and challenges of incorporating both family-structured data and repeated measurements. The topics addressed by these contributions were divided into 4 categories: (a) h^2^, (b) drug treatment response, and (c) targeted versus epigenome-wide and (d) family versus unrelated data sets. In addition, 2 of these contributions also focused on quality control of the data.

**Table 1:** GAW20 quality control and statistical models, data sets, and software used by this group

Contribution	Phenotype	Normalization	h^2^	Covariates	CpG probes	Model(s)	Software
Almeida et al. [22]	HDL	Inverse	Pre and post fenofibrate HDL and CpG sites	20 PCs	Epigenome-wide	VC-LME	SOLAR
Canty and Paterson [20]	TG, QC	Probe type strata	–	4 PCs Difference in TGs	Epigenome-wide	Standard linear model (t-test)	
Fernandez-Rhodes et al. [25]	Metabolic syndrome	Type II probes	Metabolic syndrome, 4 CpG sites	age, sex, SNPs, center, smoking, PCs	4 CpG sites	VC-LME	SOLAR
LeBlanc et al. [21]	QC only	BMIQ	Used breeding values from a heritability model	Age, sex, SNPs	Epigenome-wide	Bayesian LME	R-INLA
Lim et al. [23]	TG	BMIQ		Age, sex, study, center, smoking, 10 PCs	14,850 CpG sites showing	LME	WGCNA, missmethyl
Nustad et al. [24]	TG, HDL	BMIQ	P-to-e and post fenofibrate, TG HDL and CpG sites	Age, sex	Epigenome-wide	Bayesian LME	R-package INLA
Yu et al. [26]	TG	–		Age, sex, study center, smoking status, HDL	349,755 CpG sites	GEE	R 3.2

All contributions in this group used the GAW20 real data set

*BMIQ* beta-mixture quantile normalization method for correcting probe design bias, *GEE* generalized estimating equation, *HDL* high-density lipoprotein, *h*^*2*^ denotes heritability and indicates if the paper has estimated this quantity, *INLA* integrated nested Laplace approximation, *PC* DNA methylation-derived principal component and indicates this study employed PCs as covariates in their analysis, *QC* quality control, *SOLAR* sequential oligogenic linkage analysis routines, *SNP* single-nucleotide polymorphism, *TG* triglyceride, *VC-LME* variance component linear mixed effect, *WGCNA* weighted gene coexpression network analysis

## Methods

### GAW20 data

GAW20 data included a real and simulated data set on 200 replicates of the real data phenotypes and CpG sites for 2 time points, before and after 3 weeks of daily treatment with a lipid-lowering drug (fenofibrate). All 7 contributions summarized here used the GAW20 real data set. The real data set was provided by the Genetics of Lipid Lowering Drugs and Diet Network (GOLDN) study and included EWAS and genome-wide association genotypes from 188 extended families from Minnesota and Utah [15]. DNA methylation in CD4+ cells was measured using the 450 K Infinium array for 463,995 CpG sites and was available for 995 and 530 individuals pretreatment and posttreatment, respectively. Phenotype information included sex, age, recruitment center, smoking status, and blood lipid levels, and was available both pretreatment and posttreatment for 818 and 861 individuals for TG and high-density lipoprotein (HDL), respectively.

### EWAS quality control

An important consideration for EWAS studies are quality control (QC) and normalization of the CpG sites. Proper QC helps detect bias and potential technical confounders, and is essential in both cross-sectional and longitudinal studies, making sure phenotypic data between time points are comparable.

The Illumina Human Methylation 450 K array (Illumina, San Diego, CA USA), uses 2 different chemistries to detect DNA methylation. As these 2 chemistries differ in dynamic range, sensitivity, and biological annotation, the observed methylation patterns follow 2 different distributions. This is further complicated by the mixture distributions generally observed in methylation data. Multiple methods have been developed to address the issues caused by the 2 chemistries [16–19]. Two of the GAW20 contributions from this working group focused solely on QC of the EWAS data [20, 21].

Canty and Paterson [20] focused mainly on batch effects and QC using independent observations, while LeBlanc et al. [21] focused on using family structure in their QC steps. Inspection of the provided GAW20 data by both studies revealed insufficient probe-normalization, which was addressed in multiple papers submitted to GAW20, including 5 contributions from this group [20–24]. Three of these studies [21, 23, 24] used beta-mixture quantile normalization (BMIQ) [19] to normalize out probe-type effects, whereas Canty and Paterson [20] analyzed the CpG probe types in 2 separate strata. Almeida et al. [22] used inverse-normalization to convert the DNA methylation beta values to have range (−∞,∞). For the same reason, Canty and Paterson [20], Nustad et al. [24], and LeBlanc et al. [21] used the inverse logit transformation of beta values (M values) for their analysis. Two contributions from this group [25, 26] did not use any normalizations on the epigenetic data. However, because Fernández-Rhodes [25] only used Type II probes, the observed probe bias should not have affected their results.

Further inspection of the epigenetic data indicated strong batch effects for pretreatment and posttreatment, as well as evidence for sample swaps. This was clearly outlined in both the QC contributions by 2 of these GAW20 contributions [20, 21]. Batch effects in genomic studies are sometimes adjusted for by adding the principal components (PCs) in the analysis. In our GAW20 group, 4 groups [20, 22, 23, 25] adjusted for DNA methylation-derived PCs in their analysis.

The interpretation of DNA methylation-derived PCs is still unclear in EWAS, but is often taken to represent either batch effects or reflect the sample-specific cell-type composition. In Irving et al. [27], the PCs were interpreted as impurities in the CD4+ T-cell population.

### GAW20 approaches

#### Heritability

Three GAW20 contributions in this group estimated h^2^ based on the reported family relationships for either phenotypes or DNA methylation. Narrow-sense h^2^ was estimated for blood lipids [22, 24], metabolic syndrome [25], treatment effect [24], and DNA methylation [22, 24, 25]. All 3 of these contributions used a linear mixed effect (LME) model (variance component model) approach. Frequentist models [22, 25] were implemented in SOLAR (sequential oligogenic linkage analysis routines) [9], whereas a Bayesian model [24] was implemented in INLA (integrated nested Laplace approximation) for h^2^ estimates [28].

All 3 contributions [22, 24, 25] estimated h^2^ with some clinical covariates accounted for in their LME models. Two of these contributions investigated pretreatment and posttreatment h^2^ estimates epigenome-wide [22, 24], while Fernández-Rhodes [25] focused on metabolic syndrome-associated CpG sites. Almeida et al. [22] used LME to estimate h^2^ of inverse-normalized CpG sites epigenome-wide. These researchers also investigated HDL h^2^ for both pretreatment and posttreatment with and without the first 20 DNA methylation-derived PCs as covariates in their LME [22]. In addition, they calculated covariance matrices between samples based on gene-specific methylation sites. These matrices were used as an additional component in a LME, where they investigated if some of these matrices could explain a significant proportion of the HDL phenotypic variance.

Fernández-Rhodes et al. [25] estimated the h^2^ of 4 CpG sites (cg00574958, cg17058475, cg18181703, and cg06500161) previously associated with metabolic syndrome in GOLDN and other studies, focusing on building LMEs, as implemented in SOLAR [9] to account for shared genetic and environmental factors. They preprocessed the pretreatment methylation data by adjusting for the top 4 methylation PCs, as previously described to account for T-cell purity or residual batch effects [27]. Using a variance-component LME, they estimated h^2^ for metabolic syndrome and the 4 specific metabolic syndrome CpG sites in models with (a) no covariates, (b) with individual-level covariates that incorporated age, sex, and their interactions, (c) sequentially adding environmental covariates (study center, smoking status) to the (b) model. Only covariates with *p* < 0.1 were kept in their reduced model. Finally, to this reduced model, they separately added a household variance component for siblings representing “early life shared environment” and one for parents representing “later life shared environment,” and then screened for nominally significant *cis*-acting and *trans*-acting single-nucleotide polymorphisms (SNPs).

Nustad et al. [24] applied a Bayesian LME implemented in R-INLA [28] to estimate the h^2^ of TG and HDL, treatment response (change in TG and HDL from pretreatment to posttreatment) and epigenome-wide CpG sites. They performed model selection using the deviance information criterion to identify CpG sites having strong evidence of nonzero h^2^. They used BMIQ-normalized methylation on the M-scale in their analyses, and excluded SNP-associated CpG sites while accounting for age and sex.

#### Drug treatment response

Two contributions [23, 26] in this group examined the treatment effect on DNA methylation. Yu et al. [26] used a generalized estimating equation to estimate the association between log-transformed TG levels and the methylation proportion separately for the pretreatment and posttreatment of 349,755 CpG sites that were uniquely mapped to a gene. They adjusted for age, sex, study center, and smoking status in their analysis. Furthermore, they examined whether the effect of adding log-transformed HDL to the model changed the evidence for association. Using the subset of 421 individuals with methylation and lipids at both time points they also conducted a longitudinal analysis, adding a covariate fenofibrate treatment (time) and the interaction between treatment (time) and methylation proportion. Except for the added indicator covariate for drug treatment, they used the same covariates in the longitudinal modeling and conducted with and without adjustment for HDL. Lim et al. [23] restricted their analysis to 14,850 CpG sites that were nominally significant (*p* < 0.05) with log-transformed TG level at baseline methylation. For each of these sites, residuals were found from a LME accounting for family structure and covariates, such as age, sex, study center, smoking status, and 10 PCs separately pretreatment and posttreatment DNA methylation. These residuals were used to construct networks using weighted gene coexpression network analysis (WGCNA) to find modules of highly interconnected CpG sites [29]. They tested whether pretreatment modules changed more than by chance in the posttreatment modules using both the WGCNA module preservation method and generalized hamming distance [30].

#### Targeted versus epigenome-wide data

In EWAS, a proportion of DNA methylation covering the epigenome is investigated, with little regard for prior biological knowledge or reasoning. Although this is more computationally intensive, it has the ability to detect previously unknown epigenetic associations with a phenotype and generate new hypotheses about the underlying biology of complex disease.

One contribution from this GAW20 group preprocessed the epigenome-wide methylation data for cell purity or residual batch effects, but then performed a targeted analysis [25] that focused on 3 genes (*CPT1A*, *SOCS3* and *ABCG1*) previously reported to be associated with metabolic syndrome [31–35]. All other GAW20 contributions from this group applied an epigenome-wide hypothesis-generating approach where the entire epigenome was interrogated. However, 2 of these contributions implemented data-driven approaches to reduce the final number of analytic tests conducted. In their network analysis, Lim et al. [23] used a reduced data set consisting of 14,850 CpG sites that showed a nominal association of log-transformed TG with pretreatment with fenofibrate DNA methylation. Almeida et al. [22] reduced the methylation data to the gene-specific CpG sites in their search for gene-specific methylation that could explain a significant proportion of the observed HDL h^2^.

#### Family versus unrelated

Six of 7 scientific contributions in our GAW20 group used the known pairwise family relationships in their analyses [21–26]. These contributions included pedigree information that described the expected proportion of shared genetic information between extended family members. The family information was used either to estimate h^2^ or genetic values for various traits [21, 22, 24, 25] or to model the dependency between individuals in drug treatment response models [23, 26].

A single GAW20 contribution from this working group used a customized unrelated data set [20]. These authors randomly selected 1 individual from each pedigree and looked for the differences in both the mean and variability of DNA methylation between pretreatment and posttreatment.

## Results

### Heritability

Almeida et al. [22] demonstrated that epigenome-wide DNA methylation h^2^ estimates differed between pretreatment and posttreatment, with higher h^2^ estimates pretreatment. When these authors included the first 20 DNA methylation-derived PCs as covariates, the pretreatment and posttreatment h^2^ distributions were similar, with reduced h^2^ estimates. In their analysis of gene-specific methylation sites that together could explain a proportion of the HDL phenotypic variance, they did not identify any significant associations.

Fernández-Rhodes et al. [25] reported metabolic syndrome h^2^ estimates in various LMEs they tested accounting for fixed covariates; including age, sex, study center, smoking status, and SNPs, along with an additional random effect representing either early or late life shared household environment. Metabolic syndrome h^2^ estimates after accounting for these random effects ranged from 0.24 to 0.46, but the estimates were not significantly different from the model including only significant fixed effects (0.43). Early life shared environment tended to decrease the estimated h^2^ while late life shared environment had the opposite effect. These authors employed a similar LME strategy for estimating DNA methylation h^2^ at a priori*-*identified 4 CpG sites previously associated with metabolic syndrome, and observed that the resulting CpG h^2^ estimates were also robust to the LME structure.

Nustad et al. [24] estimated pretreatment h^2^ for HDL (0.48) and TG (0.61) using a Bayesian approach. Their h^2^ estimates are similar to previous frequentist estimates [36] for these phenotypes, but show large uncertainty. They also identified that response to treatment was weakly heritable. For genome-wide methylation h^2^, they reported h^2^ followed a 2-group mixture model with some proportion of CpG sites having nonzero h^2^ and the remaining CpG sites following a right-skewed unimodal distribution. The mixture proportion differed remarkably pretreatment to posttreatment (zero proportion was approximately 5% vs 57%, respectively). For the remaining CpG sites with strong evidence for nonzero h^2^, the mean, median, and interquartile range were 0.33, 0.31, and 0.16 pretreatment and 0.36, 0.34, and 0.20 posttreatment.

### Drug treatment response

Yu et al. [26] found 23 CpG sites that were significantly associated with TGs in the pretreatment data after Bonferroni correction and found 13 such sites in the posttreatment data. Only 1 CpG site (cg19003390 in the *CPT1A* gene on chromosome 11) was consistently found to be associated in both data sets, and with or without adjustment for HDL. In the longitudinal analysis, 6 significant interactions were found either with or without adjustment for HDL, however, only 1 CpG site (cg20354777 in *SPSB4* on chromosome 5) showed a significant interaction in the same direction irrespective of adjustment for HDL. All other significant interactions were either significant with or without HDL adjustment but not in both models. The network analysis approach used by Lim et al. [23] resulted in 6 significant modules of at least 30 CpG sites, but the vast majority of probes (14,049) examined did not belong to any of these modules. Using both the module preservation and generalized Hamming distance methods, they found that 3 of these 6 modules had topological differences between the pretreatment and posttreatment networks. The smallest module found (44 CpG sites) was also the most different between the 2 time points using the WGCNA preservation statistic. Most of the moderate to high correlations found in the pretreatment discovery set were absent in the posttreatment module. This was also seen in the other 2 modules showing evidence of differential structure.

### Quality control, family vs unrelated

The application of h^2^ estimates represents a potential novel QC procedure for EWAS data and this methodology was explored by 3 contributions in our GAW20 workgroup [21, 22, 24]. CpG sites are responsive epigenetic elements and it is reasonable to expect that their majority present low to moderate h^2^ estimates [12, 13].

Canty and Paterson [20] conducted paired t-tests to examine changes in methylation between the pretreatment and posttreatment data sets. To avoid any issues from family structure, they used a sample size of 140 individuals, each randomly chosen from a different family. They found that almost one-third of CpG sites (149,396 out of 463,995) had a significant difference after Bonferroni correction. The significant sites were uniformly distributed across the entire genome. Methylation generally increased for Infinium Type I probes and generally decreased for Type II probes. There were also 9986 CpG sites showing a significant difference in variability between pretreatment and posttreatment, generally showing a decrease in variability after treatment. These results are from models that did not use any covariates. When the difference in TG levels was included as a covariate, fewer significant differences in methylation were found (26,371), but still many more than would be expected.

LeBlanc et al. [21] also conducted a paired t-test as a QC step to investigate the mean differences between pretreatment and posttreatment methylation. Approximately 300,000 (enhanced by family correlation) CpG sites were found to be significantly different (*p* < 0.05), indicating large differences between the time points. After BMIQ normalization, the signal dropped to approximately 240,000, indicating a missing probe-type normalization gave rise to an increased false-positive rate genome wide. In addition, 13 samples were found to be possible samples swaps based on comparison of SNP-regulated CpG methylation values pretreatment and posttreatment, breeding value correlation between pretreatment and posttreatment methylation estimated from a h^2^ model and methylation-inferred gender. Of these samples, 11 were found to be wrongly labeled in the posttreatment.

## Discussion

As with previous GAW workshops that have investigated longitudinal data, a direct comparison of results between each contribution summarized here is made difficult by the variability in phenotypes and analytical approaches implemented (see Table 1). However, there are some novel insights, strengths, and potential limitations of these statistical approaches that can be discussed. Additionally, these papers highlight some important opportunities for further exploration in the development of statistical methodologies for understanding epigenome-wide DNA methylation patterns.

**Fig. 1:**
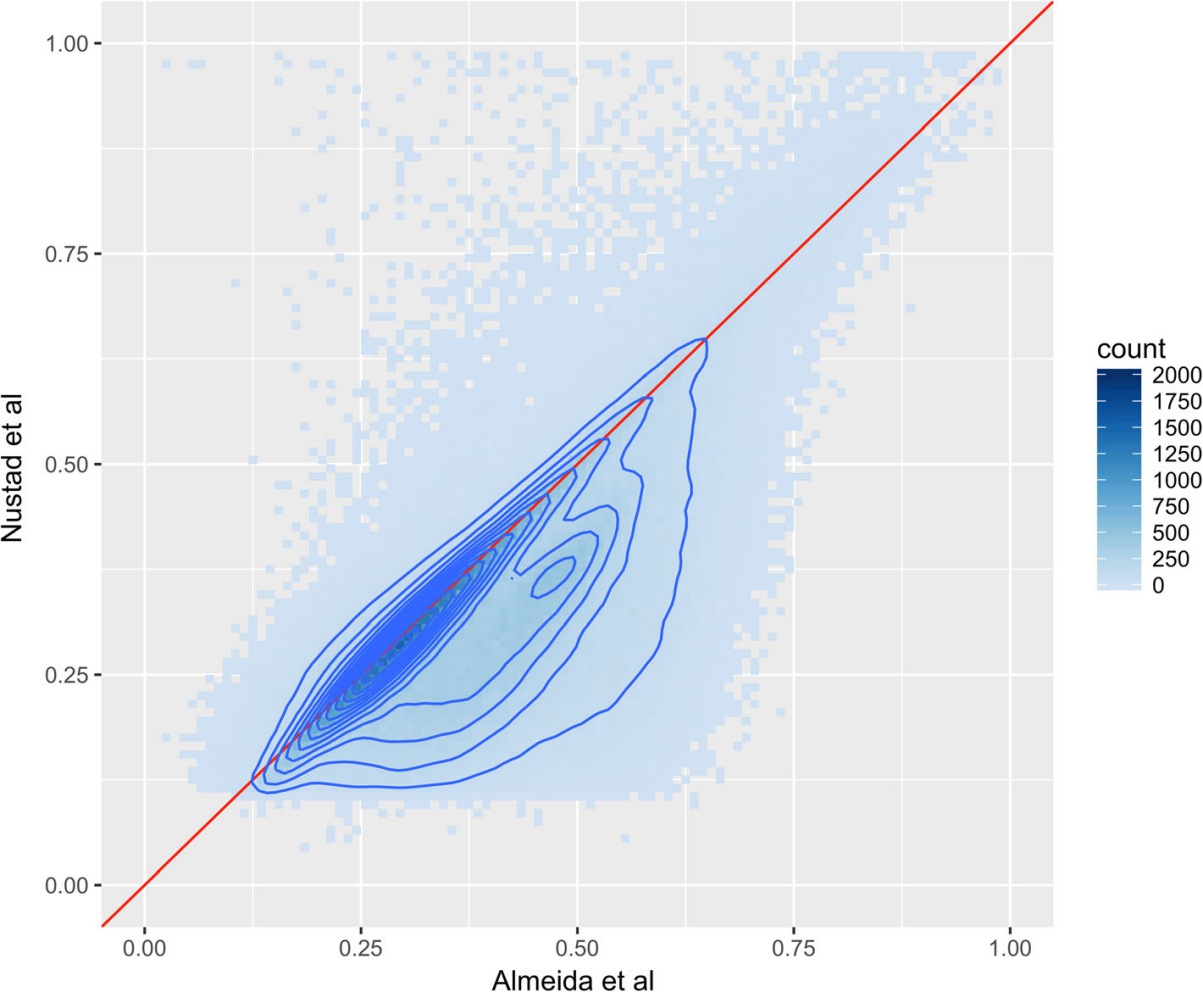
The figure shows a comparison of pretreatment DNA methylation heritability estimates from Nustad et al. [24] and Almeida et al. [22]. Each dot represents a 0.01 × 0.01 square with the color indicating the number of estimates that fall within the square. The red line is the 1-to-1 line, while the dark blue contour lines present the estimated 2-dimensional density. The displayed heritability estimates are those that passed the model selection step in Nustad et al. [24]

Two contributions directly addressed the issue of QC in the provided GAW20 EWAS data. Canty and Paterson [20] customized a reduced set of individuals, whereas LeBlanc et al. [21] used the family-based data. Both identified a huge signal of differences between the pretreatment and posttreatment DNA methylation, which differences were uniformly distributed across the genome [20], and argued for the possibility of batch effects. Although not correcting all the expected false positives between pretreatment and posttreatment, some of these differences were the result of missing normalization of Type I and Type II probe chemistries in both data sets independently, as shown in LeBlanc et al. [21] (Fig. 1).

**Fig. 2:**
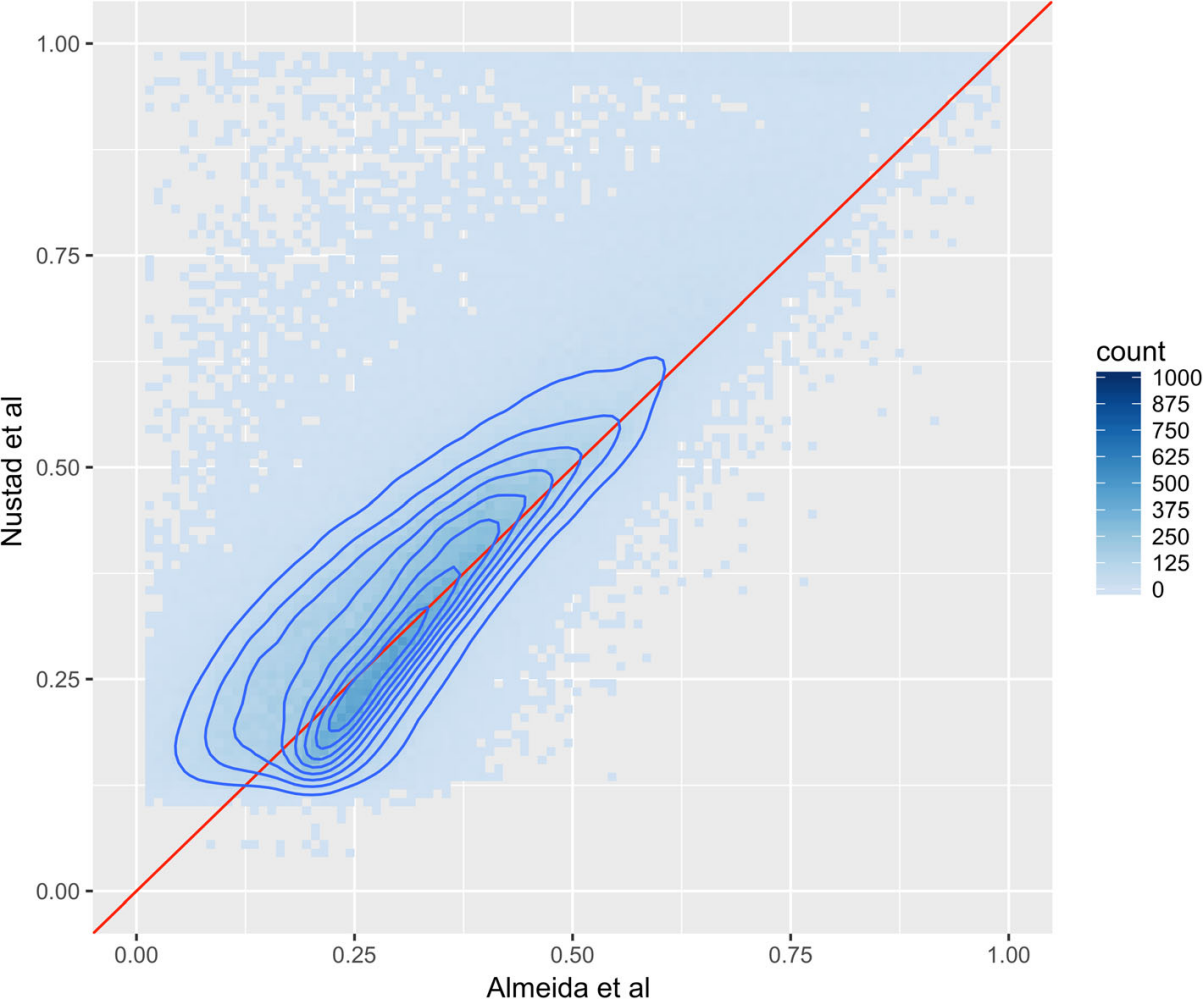
The figure shows a comparison of posttreatment DNA methylation heritability estimates from Nustad et al. [24] and Almeida et al. [22]. Each dot represents a 0.01 × 0.01 square with the color indicating the number of estimates that fall within the square. The red line is the 1-to-1 line, while the dark blue contour lines present the estimated 2-dimensional density. The displayed heritability estimates are those that passed the model selection step in Nustad et al. [24]

Both Almeida et al. [22] and Nustad et al. [24] have established epigenome-wide h^2^ estimates of pretreatment and posttreatment CpG sites. Almeida et al. [22] employed a commonly used frequentist approach implemented using SOLAR [9], while Nustad et al. [24] used a Bayesian approach called INLA [28]. Figure 1 shows the h^2^ estimates from pretreatment DNA methylation measurements from these studies, and Fig. 2 compares h^2^ estimates from posttreatment DNA methylation. The density curve indicates that the majority of measurements are close to the diagonal, indicating that the h^2^ estimates between the 2 studies are similar. The tail in Fig. 1 of the 2-dimensional density indicates that Almeida et al. [22] has a small trend toward higher h^2^ estimates than do Nustad et al. [24]. The Pearson correlation between the estimates are 0.72, indicating a high correlation between the vectors of estimates. Figure 2 indicates an opposite trend as for the pretreatment methylation, namely that Nustad et al. [24] has a small trend toward higher h^2^ estimates than Almeida et al. [22]. The Pearson correlation for the posttreatment h2 estimates is 0.82, indicating a high correlation. The correlation between the h^2^ estimates is higher for posttreatment than for pretreatment DNA methylation measurements. However, because these correlation estimates are based on the number of CpG sites that passed the model selection step, the number of CpG sites evaluated is different. The number for pretreatment is 425,791, while for posttreatment the number is 199,027. The difference in amount of nonzero h^2^ estimates could be caused by an induced familial batch effect in the pretreatment methylation data suggested by Almeida et al. [22], a loss in signal caused by sample swaps in the posttreatment methylation data suggested by LeBlanc et al. [21], or both. With an opposite general effect between the frequentist [22] and the Bayesian [24] approach in pretreatment versus posttreatment methylation, it is hard to draw any conclusion regarding comparisons of strengths and limitations of these methods. This calls for further analysis and simulation studies of the 2 approaches.

The GAW20 data did not contain information regarding shared household and dietary aspects, such that the resulting h^2^ estimates of CpG sites may represent an overestimation. However, Fernández-Rhodes et al. [25] used a novel approach to try and decompose the amount of h^2^ resulting from shared genetic and environmental factors. To account for shared early life environment, they included a random effect for siblings or half-siblings who were within 15 years of each other. They also included a random effect for parent pairs to model the shared later life environmental exposures. By including these effects for early and later life shared environment, the h^2^ estimates became more stable but did not change significantly.

Two contributions investigated drug response pretreatment and posttreatment [23, 26]. Yu et al. [26] identified differences in CpG sites pretreatment and posttreatment that might alter TG concentrations, partially through altering DNA methylation. However, the CpG sites identified pretreatment and posttreatment differed markedly. These findings suggest the existence of moderation effects of DNA methylation (or drug-methylation interactions) on TG. A total of 11 methylation sites showed substantial interaction effects with or without HDL adjustment when both pretreatment and posttreatment data were analyzed [37]. Furthermore, the interaction between the *SPSB4* gene and fenofibrate was significant regardless of HDL adjustment, suggesting this interaction effect was independent of HDL. They also conducted interaction studies and found that the association of TG with epigenetic data differed by HDL adjustment, implying that TG and HDL potentially share some epigenetic processes which warrant further investigation. Lim et al. [23] explored the relationship between TG-associated DNA methylation and fenofibrate treatment in a network framework and detected 6 subnetworks using pretreatment methylation probes. They identified 3 differentially methylated posttreatment modules using both the module preservation and the generalized Hamming distance method. Enrichment analysis revealed that some were comprised of genes involved in phospholipid metabolism, which may provide insight into the effect of treatment on methylation and TG levels. However, they could not conclude that fenofibrate induced these epigenetic alterations.

A major limitation for the GAW20 data is that treatment and time are completely confounded, which will likely attenuate the possibility to detect treatment effects on DNA methylation. Nevertheless, it may have been possible to better address this by normalizing the data from the 2 time points jointly. Unfortunately, the raw data to do this were not available. A strength for these contributions is that they suggest several improvements for estimating h^2^ in pedigrees, by attempting to correct for shared environment and by using a model selection step to access the evidence for nonzero h^2^.

## Conclusions

In this paper, we summarize 7 GAW20 contributions applying novel or existing statistical methods for epigenome-wide DNA methylation data for 2 time points pretreatment and posttreatment with fenofibrate. Despite the heterogeneous nature of these analytical approaches, this GAW20 working group was able to come to these conclusions: (a) QC measures are an important consideration for EWAS studies that are investigating multiple time points or repeated measurements; (b) comparison of h^2^ estimates between time points for individual CpG sites is a useful QC measure for DNA methylation studies; (c) drug intervention demonstrated strong epigenome-wide DNA methylation patterns across the 2 time points; and (d) new statistical methods are required to account for the environmental contributions of DNA methylation across time. The demonstrated diversity and strategies applied from this GAW20 working group show that several statistical approaches are appropriate for investigating repeated measurement data. Although certain methodological commonalities existed between these contributions, the diversity of approaches did not allow for direct comparison across all 7 of these GAW20 contributions. However, it is apparent from these contributions that numerous opportunities exist for the implementation and analysis of repeated measurement data in EWAS.

## Abbreviations

BMIQ: Beta-mixture quantile normalization
CpG: Cytosine-phosphate-guanine
EWAS: Epigenome-wide association studies
GAW20: Genetic Analysis Workshop 20
GEE: Generalized estimating equations
GOLDN: Genetics of Lipid Lowering Drugs and Diet Network
h^2^: Narrow-sense heritability
HDL: High-density lipoprotein
INLA: Intergrated nested Lapace approximation
LME: Linear mixed effect
PC: Principal components
QC: Quality control
SNPs: Single nucleotide polymorphisms
SOLAR: Sequential oligogenic linkage analysis routines
TG: Triglyceride
WGCNA: Weighted gene coexpression network analysis
